# EGFR G796D mutation mediates resistance to osimertinib

**DOI:** 10.18632/oncotarget.17913

**Published:** 2017-05-16

**Authors:** Di Zheng, Min Hu, Yu Bai, Xuehua Zhu, Xuesong Lu, Chunyan Wu, Jiying Wang, Li Liu, Zheng Wang, Jian Ni, Zhenfan Yang, Jianfang Xu

**Affiliations:** ^1^ Department of Medical Oncology, Shanghai Pulmonary Hospital, Tongji University Medical School, Shanghai, China; ^2^ IMED Asia, AstraZeneca, Shanghai, China; ^3^ Research and Development Information, AstraZeneca, Shanghai, China; ^4^ Department of Pathology, Shanghai Pulmonary Hospital, Tongji University Medical School, Shanghai, China

**Keywords:** EGFR, NSCLC, osimertinib, drug resistance, G796D

## Abstract

Osimertinib is an effective third-generation epidermal growth factor receptor (EGFR) tyrosine kinase inhibitor (TKI) approved in multiple countries and regions for patients with EGFR T790M mutation-positive non-small cell lung cancer (NSCLC). Despite impressive initial tumor responses, development of drug resistance ultimately limits the benefit of this compound. Mechanisms of resistance to osimertinib are just beginning to emerge, such as EGFR C797S and L718Q mutations, BRAF V600E and PIK3CA E545K mutations, as well as ERBB2 and MET amplification. However, a comprehensive view is still missing. In this study, we presented the first case of Chinese NSCLC patient who developed resistance to osimertinib, and discovered *de novo* EGFR G796D mutation as a potential mechanism. Our findings provided insights into mechanisms of resistance to osimertinib and highlighted tumor heterogeneity and clonal evolution during the development of drug resistance.

## INTRODUCTION

Epidermal growth factor receptor (EGFR) activating mutations (e.g., L858R and exon 19 deletion) account for 30–60% of non-small cell lung cancer (NSCLC) cases in Asia [1–3]. NSCLC patients with EGFR activating mutations respond to first- and second-generation EGFR tyrosine kinase inhibitors (TKIs) [4–13]. However, drug resistance inevitably develops, with ∼60% of events attributing to a secondary EGFR T790M gatekeeper mutation [14–16]. Osimertinib is an oral, irreversible, mutant-selective third-generation EGFR-TKI developed against NSCLC bearing EGFR activating mutation and T790M [17–19]. In the AURA and AURA3 studies, osimertinib was highly active in lung cancer patients with T790M mutation who had progressed during prior therapy with EGFR-TKIs [20, 21]. Among T790M-positive NSCLC patients, the median progression-free survival (PFS) was significantly longer with osimertinib than with platinum therapy plus pemetrexed (10.1 *vs*. 4.4 months; hazard ratio, 0.30; *P* < 0.001), and the objective response rate (ORR) was significantly better with osimertinib than with platinum therapy plus pemetrexed (71% *vs*. 31%; odds ratio, 5.39; *P* < 0.001) [21].

So far, only a few studies have been performed to understand potential mechanisms of resistance to osimertinib. EGFR C797S mutation has been demonstrated as a principle mechanism of acquired resistance [22, 23], presumable through abolishing the covalent binding between osimertinib and C797 residue. In addition, there are a limited number of case reports detailing the identification of EGFR L718Q, BRAF V600E and PIK3CA E545K mutations, as well as ERBB2 and MET amplification [23–26]. Little is known about alternative resistance mechanisms, the prevalence of each type of mechanisms, and the ethnic differences in resistance profiles. In this study, we present a case report on the first Chinese osimertinib NSCLC patient. We closely monitored the disease course of the patient, prospectively collected pretreatment and post-disease progression plasma specimens, and identified a standalone EGFR G796D mutation as a new resistance mechanism to osimertinib.

## RESULTS

### Medical history of patient246

**Figure 1:**
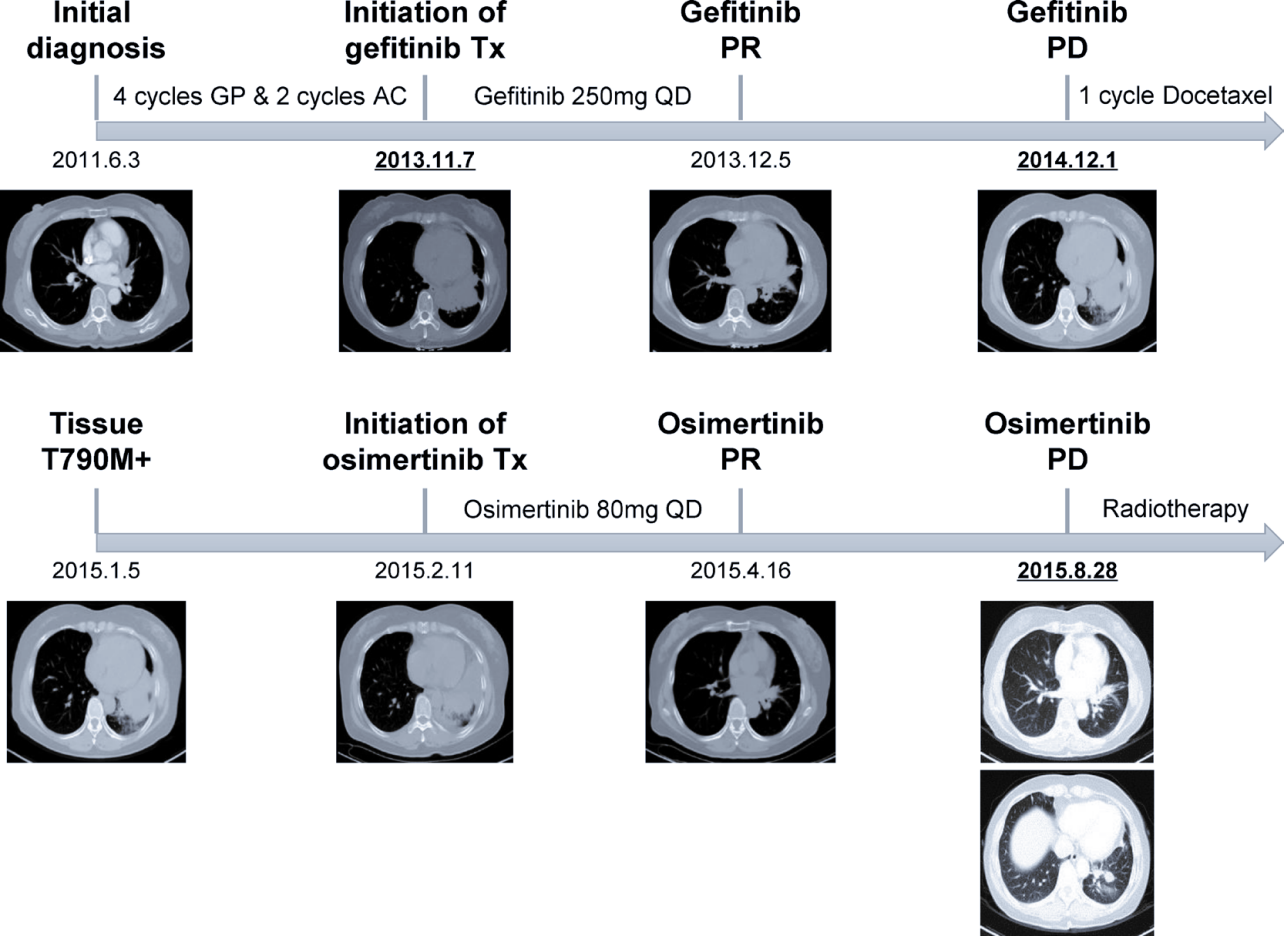
Medical history of patient246 The patient first underwent multiple lines of chemotherapy and gefitinib treatment. After the resistance biopsy was diagnosed as T790M-positive, she received osimertinib through “named patient use”. Abbreviations: GP, gemcitabine/cisplatin; AC, pemetrexed/carboplatin; Tx, treatment; PR, partial response; PD, progressive disease. The three time points of plasma sample collection are underlined and bolded.

Patient246 was a 56 year-old female diagnosed as stage IV lung adenocarcinoma (T4N2M1b), with multiple distant metastasis lesions including brain and bone (Figure 1, Supplementary Figure 1). She had received multiple lines of chemotherapy including 4 cycles of gemcitabine/cisplatin and 2 cycles of pemetrexed/carboplatin for 2 years and 5 months in total. After disease progression, she switched to gefitinib and showed a partial response within 1 month of treatment. At 13-month post-gefitinib, she relapsed and EGFR T790M mutation was detected from biopsy of progressed primary lesion (Figure 1). Through special approval of “named patient use” due to unavailability of osimertinib in Chinese market at the time, she became the first patient to receive osimertinib in China. After 6 weeks of treatment, scans demonstrated a partial response. However, she developed systemic progressive disease (PD) at ∼6.5-month post-osimertinib and switched to radiotherapy subsequently (Figure 1).

### Identification of EGFR G796D mutation from plasma samples

**Figure 2:**
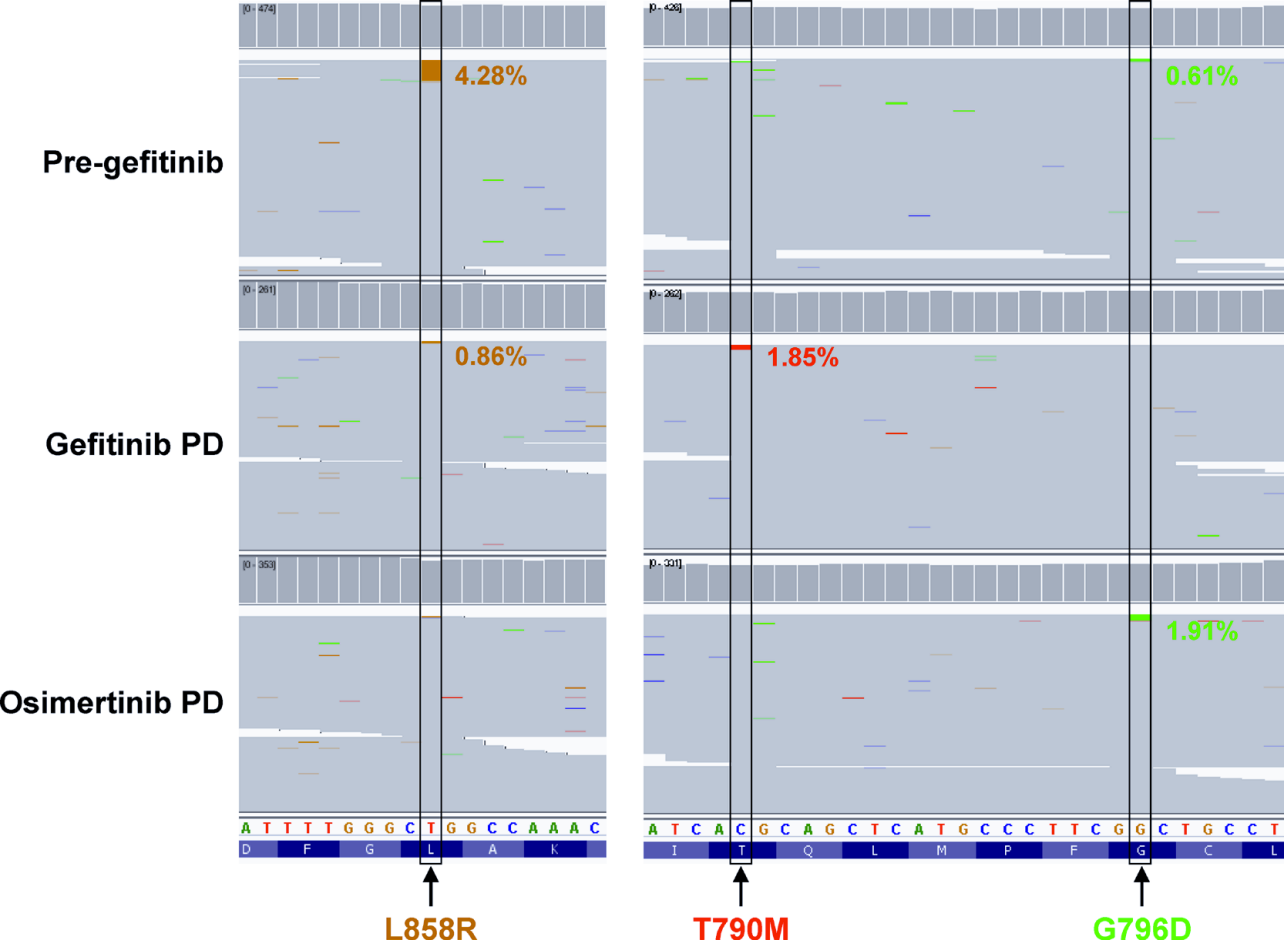
Dynamics of EGFR L858R, T790M and G796D mutations IGV view of variant calls covering EGFR L858, T790 and G796 residues from panel sequencing of plasma samples at indicated time points. G796D mutation increased from 0.61% of reads at pre-gefitinib to 1.91% upon osimertinib PD, while T790M acquired at gefitinib resistance was cleared by osimertinib.

To understand the mechanisms underlying resistance to osimertinib in patient246, we collected and profiled her plasma samples from three time points – pre-gefitinib, gefitinib PD and osimertinib PD (Figure 1). Using next-generation panel sequencing, we identified EGFR L858R mutation with mutant allele frequency (MAF) of 4.28% in the pre-gefitinib plasma sample (Figure 2). Interestingly, low MAF (0.61%) of EGFR G796D was also detected at this point. After gefitinib PD, T790M emerged to MAF of 1.85%, consistent with tissue testing results. Upon osimertinib PD, both L858R and T790M became undetectable, however, G796D increased to MAF of 1.91% (Figure 2). As control, none of the three types of mutations were detected in whole blood. These data suggested that G796D was a *de novo* mutation likely involved in resistance to osimertinib. The different dynamics of L858R, T790M and G796D during treatment journey also implicated that G796D might be a standalone mutation independent of L858R and T790M.

### EGFR G796D mutation as the resistance mechanism to osimertinib

**Figure 3:**
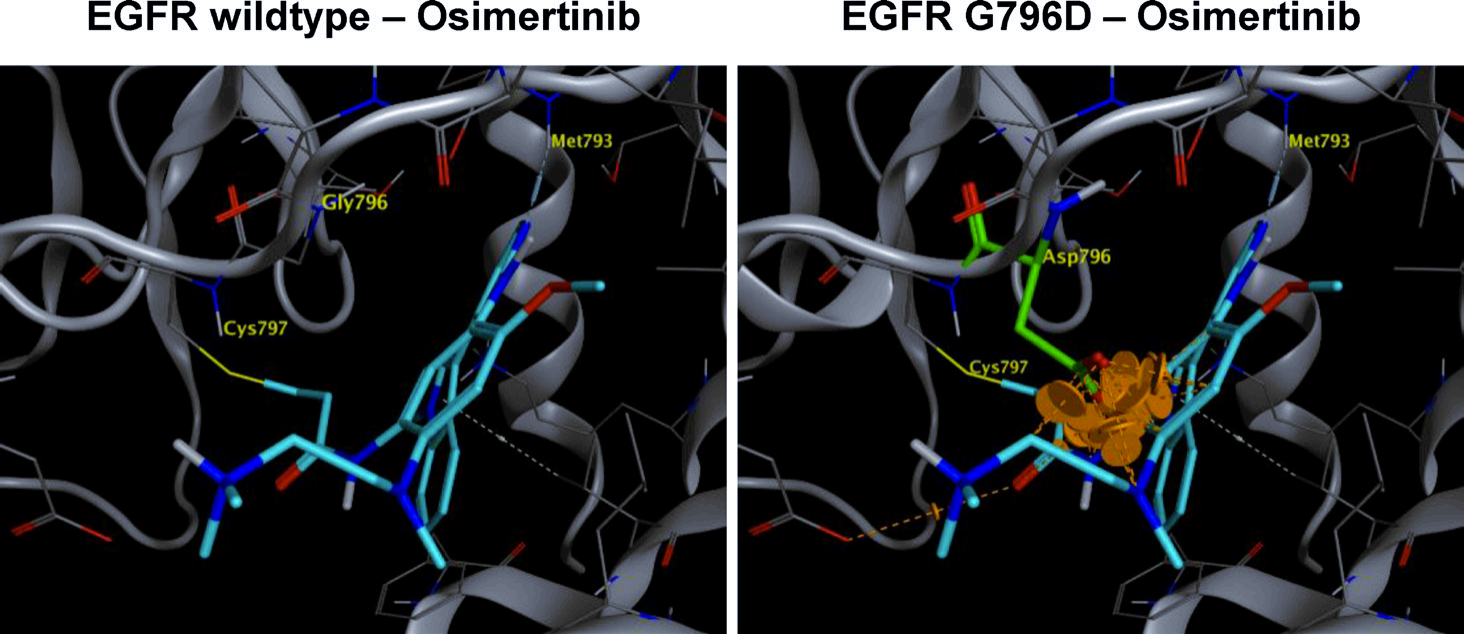
Structural modeling of EGFR G796D in complex with osimertinib G796D mutation is expected to sterically interfere with binding of osimertinib. The hydrophilic side chain CH2COOH of mutated D796 residue will bump into the greasy core of osimertinib, which would either push the inhibitor out of the current binding mode, or distort the loop and affect the hinge binding. Either case could lower the binding affinity of osimertinib. The orange disks indicate the clashes between the side chain of the mutated reside and the core of osimertinib.

G796 is located adjacent to C797 residue, where osimertinib forms covalent bond with EGFR. Thus we hypothesized that G796D could drive resistance to osimertinib by interfering with osimertinib-EGFR interaction. Indeed, structural modeling of EGFR kinase domain in complex with osimertinib showed that the side chain of mutated D796 residue would clash into the molecular surface of osimertinib, lead to a steric and energetic repulsion, and result in the loss of binding affinity (Figure 3).

**Figure 4:**
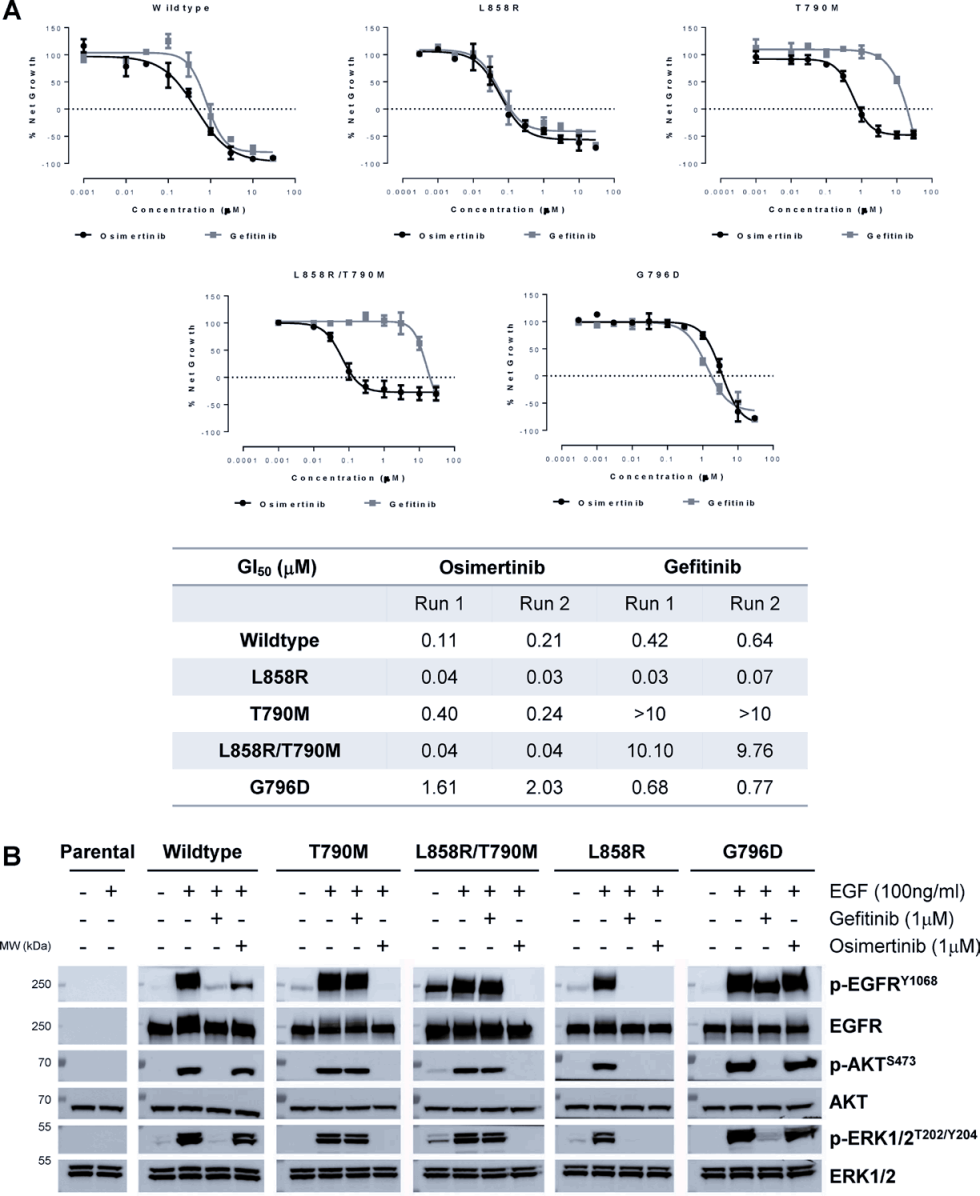
EGFR G796D confers resistance to osimertinib (**A**) Upper: Ba/F3 cells harboring various EGFR mutants were treated with osimertinib or gefitinib at indicated concentrations. Viable cells were measured after 72 h of treatment and cell proliferation was calculated as % Net Growth = (G_day3, inh_ - G_0_)/(G_day3, ctl_ - G_0_) × 100%. Experiments were repeated twice, with mean ± S.D. plotted at each concentration. The curves were fitted using a nonlinear regression model with a sigmoidal dose response. Lower: GI_50_ (μM) of the two independent runs. (**B**) Ba/F3 cells expressing various EGFR mutants were pre-incubated with 1 μM gefitinib or osimertinib for 2 h followed by stimulation of EGF for 10 min in serum-free medium. Cell extracts were immunoblotted to detect phosphorylated or total EGFR, AKT and ERK1/2 levels.

To confirm that G796D could induce resistance to osimertinib, we generated stable lines of Ba/F3 cells carrying various EGFR mutants (Supplementary Figure 2). IL3-independent growth assay suggested that G796D was likely a mild oncogenic driver comparing to L858R and L858R/T790M mutations (Supplementary Figure 3). Osimertinib inhibited the growth of L858R or L858R/T790M lines with GI_50_ of 30–40 nM, but GI_50_ increased to 1.5–2 μM in G796D mutant line, with about 50-fold shift (Figure 4A). Moreover, p-EGFR level of G796D mutant line, and its downstream p-AKT and p-ERK1/2 levels were not modulated by osimertinib up to 1 μM concentration (Figure 4B). These cellular functional results suggested that G796D mutation rendered the resistance to osimertinib.

## DISCUSSION

Plasma cell-free DNA (cfDNA) samples have been shown to contain tumor-specific genomic alterations and utilized to dynamically monitor tumor response and relapse [27, 28]. Comparing with tissue testing, plasma testing is often associated with lower sensitivity but on the other hand, can circumvent the challenges in obtaining re-biopsy specimens and “false-negatives” resulted from tumor heterogeneity. The abundances of mutations in cfDNA are correlated with tumor burden, stage and metastasis status [29, 30]. And several studies have demonstrated that quantitative measurement of mutation abundances in cfDNA could be used to accurately reflect clinical response and emerge of resistance [31–33]. In this study, we used cfDNA to understand the resistance mechanisms of osimertinib.

**Figure 5:**
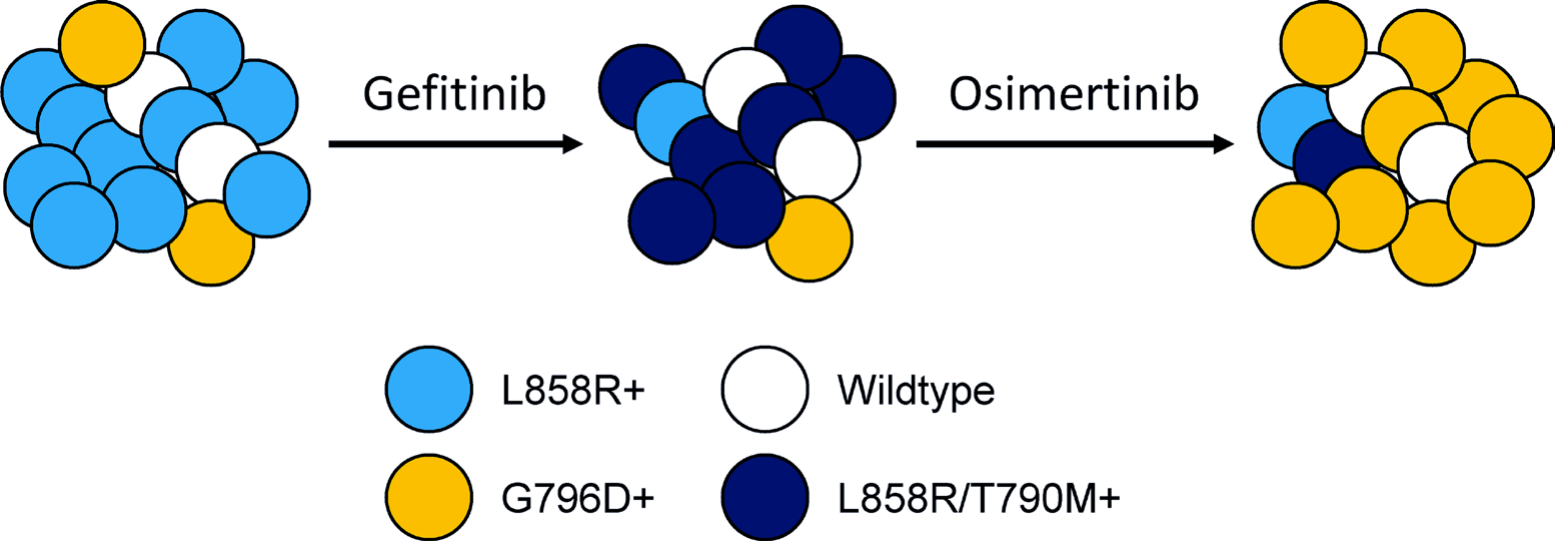
Tumor heterogeneity and clonal evolution A conceptual model showing that the tumor was genomically heterogeneous, comprising of both L858R-positive and G796D-positive populations, with the former as the major clone. Upon gefitinib resistance, L858R-positive cells acquired the secondary T790M mutation and became the dominant driver of tumor growth. These L858R/T790M-positive tumor cells were then inhibited by osimertinib, whereas pre-existing G796D-positive clones, which are resistant to osimertinib, gradually made up the majority of the population.

Our dynamic genomic analysis of serial cfDNA samples from patient246 at time points of pre-gefitinib, gefitinib PD and osimertinib PD discovered EGFR G796D mutation as a new resistance mechanism to osimertinib. The MAF of G796D in cfDNA samples increased from 0.61% at pre-gefitinib to 1.91% upon osimeritinib PD. Meanwhile, the acquired T790M mutation following gefitinib PD was cleared to the undetectable level by osimeritinib, which is consistent with the reported data in some patients receiving second-line treatment of osimertinib or rociletinib, another third-generation EGFR-TKI [22, 23, 26, 34, 35]. This dynamic switch between G796D and T790M mutations suggested a clonal evolution event which featured diminishment of T790M-positive clones and outgrowth of pre-existing G796D-positive clones under selection pressure of osimertinib (Figure 5).

Previously, G796C/D/R were demonstrated to render resistance to erlotinib and CI-1033 in Ba/F3 cell system through a random mutagenesis screening approach [36]. The impact was more dramatic on CI-1033, a pan-ErbB inhibitor that forms a covalent bond with EGFR C797 residue [37]. The GI_50_ increased 10- and 100-fold for erlotinib and CI-1033, respectively, in G796R mutant line compared with Ba/F3 cells expressing wildtype EGFR [36]. G796D could induce resistance to osimertinib by interfering with osimertinib-EGFR interaction due to steric hindrance around C797 residue, the covalent binding site for the inhibitor. We noted that G796D is resistant to gefitinib as well as erlotinib (Supplementary Figure 4), albeit to a less extent. However, G796D-positive cells remained as minority of population upon gefitinib PD. We reasoned that it was probably because G796D is a milder mutation comparing to L858R/T790M. Therefore, L858R/T790M-positive population presented as the dominant force of tumor growth at gefitinib resistance, and G796D-positve cells only emerged as majority after L858R/T790M-positive clones were diminished by osimertinib (Figure 5).

To our knowledge, all the EGFR mutations previously reported to drive resistance to osimertinib or rociletinib, such as L718Q, C797S and L798I, were identified with T790M concurrently [22–24, 34], while G796D is found in the absence of T790M context. Hence this is the sole case by far of EGFR mutation-dependent mechanism causing intrinsic resistance to osimertinib. G796A/D/S mutations were reported in TKI-naive lung adenocarcinoma patients, either alone or in combination with exon 19 deletion [38–40], supporting that it can exist as a *de novo* oncogenic mutation. Consistently, in our case, G796D was identified together with L858R at pre-gefitinib, as a minor, independent clone. It remains to be seen whether G796D can also present as an acquired resistance mutation.

In conclusion, we have identified a case of Chinese NSCLC patient with intrinsic EGFR G796D mutation that led to resistance to osimertinib. The loss of T790M-positive clones and selection of pre-existing G796D-positive clones suggest that genomic heterogeneity contributes to clonal evolution which ultimately results in the emergence of drug resistance during treatment journey. Our findings highlight the needs for development of new targeted therapies and combination strategies dynamically adjusted based on real-time monitored genomic analysis throughout the entirety of disease course.

## MATERIALS AND METHODS

### Patient information

Patient246 was diagnosed with advanced NSCLC at Shanghai Pulmonary Hospital in June 2011. The study was approved by the hospital research ethics committee and the patient had signed the informed consent form. Serial plasma samples were collected at time points of pre-gefitinib, gefitinib PD and osimertinib PD. Whole blood were obtained at osimertinib PD. Tumor responses were assessed according to Response Evaluation Criteria In Solid Tumors Version 1.1 (RECIST 1.1).

### Extraction of genomic DNA and plasma cell-free DNA (cfDNA)

Genomic DNA from whole blood was extracted using QIAamp DNA Mini Kit (Qiagen, Hilden, Germany) according to the manufacturer's instructions. Plasma samples were prepared and stored as described previously [33]. cfDNA was extracted with QIAamp Circulating Nucleic Acid Kit (Qiagen) according to the manufacturer's protocol.

### Next-generation panel sequencing

Indexed Illumina NGS libraries were prepared from plasma cfDNA and fragmented genomic DNA using KAPA Hyper Prep Kit (Kapa Biosystems, Woburn, MA, USA) according to the manufacturer's protocol. Ligation was performed at 4°C overnight using adapters from SeqCap Adapter Kit (Roche NimbleGen, Madison, WI, USA). The indexed libraries were captured using a customized SeqCap EZ Choice Library (Roche NimbleGen) covering 403 genes (Supplementary Table 1). Hybrid selection, washing, recovery, amplification and purification of captured multiplex DNA samples were performed using SeqCap EZ system (Roche NimbleGen). The amplified products were then purified with QIAquick PCR Purification Kit (Qiagen), and multiplexed libraries sequenced on the Illumina HiSeq X10 platform with 150-bp paired-end reads according to the manufacturer's protocol. The raw average sequence depth was >7000× for plasma cfDNA samples and ∼2800× for the genomic DNA from whole blood.

### Bioinformatics data analysis

BWA [41] was employed for mapping the pair-end reads to human reference genome hg19, samblaster [42] for marking duplicate reads, and VarDict [43] for detection of single nucleotide variants (SNVs) and small insertions and deletions (indels). SNVs and small indels were called with the criteria of allele frequency ≥ 0.5% and at least two supporting reads after deduplication. Variant calls of interest were manually inspected in IGV [44, 45].

### Structural modeling

G796 was mutated to aspartic acid based on the optimal trajectory for the side chain using the EGFR-osimertinib co-crystal structure (PDB code: 4ZAU) and “Mutate Residue” method in Maestro (Schrodinger LLC., New York, NY, USA).

### Cell proliferation assay

Generation of Ba/F3 stable lines expressing various EGFR mutants was described in Supplementary Materials and Methods. For MTS assay, Ba/F3 cells were prepared in RPMI1640 with 10% FBS and 10 ng/ml EGF, seeded in 96-well plates at 30,000 cells per well, and incubated overnight. The next day, compounds at different concentrations were added to the assay plates, and cells were incubated for additional 72 hours. Following compound treatment, 20 μl/well of CellTiter 96^®^ AQueous One Solution Reagent (Promega, Madison, WI, USA) was added, incubated at room temperature for 2 hours, then 25 μl/well of 10% SDS was added to stop the reaction. The absorbance was measured at 490 nm wavelength using 650 nm as reference on Tecan Spark 20M (Tecan Group Ltd., Zurich, Switzerland). The data was processed and plotted using GraphPad Prism 6 (GraphPad Software Inc., San Diego, CA, USA).

### Modulation of signal pathways

Ba/F3 cells harboring various EGFR mutants were starved in RPMI1640 for 4 hours, pre-treated with 1 uM gefitinib or osimertinib for 2 hours, and then stimulated by 100 ng/ml EGF for 10 minutes in serum-free medium. Cells were collected, washed once in cold PBS and lysed in 2×SDS lysis buffer [100 mM Tris pH6.8, 4% SDS, 20% glycerol, and 1× protease and phosphatase inhibitors (Pierce | Thermo Fisher Scientific, Waltham, MA, USA)]. The lysates were boiled at 100°C for 10 minutes, and protein concentration was quantified by BCA Protein Assay Kit (Pierce). Equal amount of protein was loaded onto SDS-PAGE gel, transferred to nitrocellulose membrane using iBolt (Invitrogen, Carlsbad, CA, USA), and subjected to immunoblotting analysis with indicated antibodies according to manufacturers' instructions.

## SUPPLEMENTARY MATERIALS FIGURES AND TABLE




