# Supplementary material for: EGFR G796D mutation mediates resistance to osimertinib
Source: Oncotarget. 2017 May 16;8(30):49671–9. doi: 10.18632/oncotarget.17913 (PMC5564797; doi:10.18632/oncotarget.17913)
Supplement: Supplementary file 1 [file oncotarget-08-49671-s001.pdf]

EGFR G796D mutation mediates resistance to osimertinib

SUPPLEMENTARY MATERIALS

MATERIALS AND METHODS

Reagents and antibodies

Compounds were provided by AstraZeneca. Recombinant human EGF was purchased from R&D Systems (Minneapolis, MN, USA), and all antibodies from Cell Signaling Technology (Beverly, MA, USA) (Catalogue number: p-EGFR^{Y1068} #2234; EGFR #4267; p-AKT^{S473} #9271; AKT #9272; p-ERK1/2$^{T202/Y204}$ #4370; ERK1/2 #9102; and GAPDH #2118).

Cell culture

Ba/F3 cells were cultured in RPMI1640 (Gibco | Thermo Fisher Scientific, Waltham, MA, USA) with 10% fetal bovine serum (FBS; Gibco) and 10% WEHI3B conditioned medium (ATCC, Manassas, VA, USA) as a source of IL-3. 293T cells were culture in DMEM (Gibco) with 10% FBS. All cells were maintained in a humidified incubator at 37°C with 5% CO2. Cell identify was confirmed by GenePrint® 10 System (Promega, Madison, WI, USA).

Generation of Ba/F3 stable lines harboring various EGFR mutants

Full-length cDNAs of human EGFR (NM005228.3) containing different mutations were generated at Shanghai Sunbio Biotechnology Co., Ltd (Shanghai, China) and confirmed by Sanger sequencing. The cDNAs of EGFR wildtype, L858R, T790M, L858R/T790M and G796D were then subcloned into PLVX-Puro lentiviral vector (Shanghai Sunbio Biotechnology Co., Ltd). The lentivirus were packaged in 293T cell by transfection of lentiviral constructs and packaging mix (Shanghai Sunbio Biotechnology Co., Ltd). Ba/F3 cells were infected by lentivirus with 5 µg/ml polybrene (Sigma-Aldrich, St. Louis, MO, USA), selected in 2 µg/mL puromycin (Invitrogen, Carlsbad, CA, USA) as stable pools, and maintained in 1 µg/mL puromycin. Expression of exogenous EGFR was confirmed by western blot at protein level and Sanger sequencing at mRNA level.

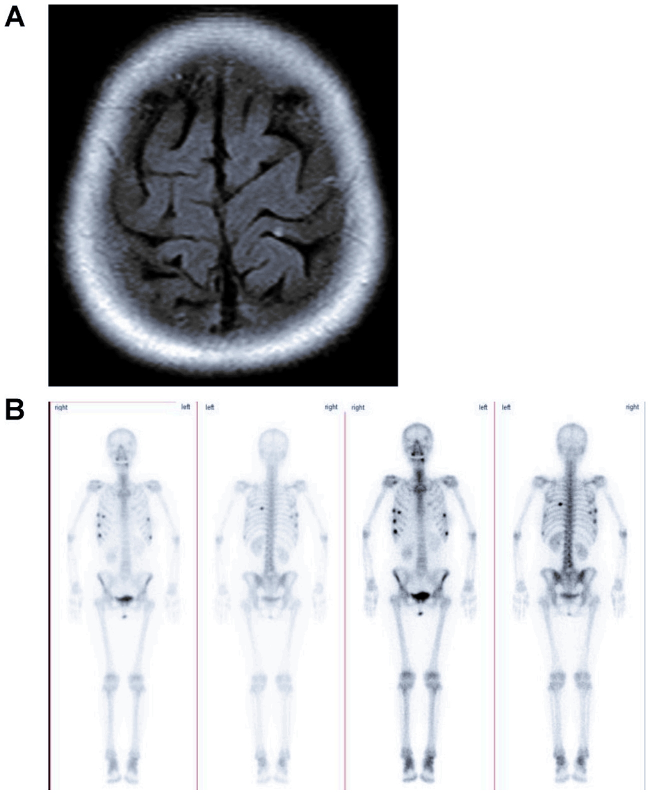

Supplementary Figure 1: Multiple metastasis lesions outside of lung in patient246 during disease course. (**A**) Magnetic resonance imaging (MRI) showed brain metastasis at initial diagnosis. (**B**) Emission computed tomography (ECT) showed bone metastasis during chemotherapy regimen before gefitinib treatment.

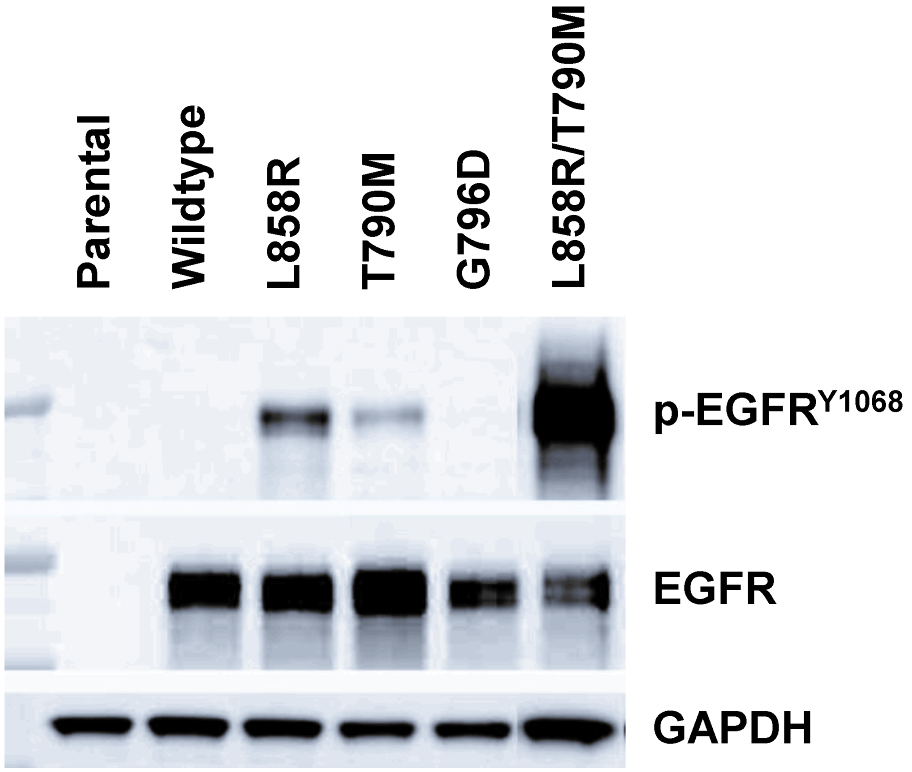

Supplementary Figure 2: Basel level of p-EGFR in Ba/F3 cells expressing various EGFR mutants. Cells were cultured in RPMI1640 + 10% FBS without IL3 nor EGF, and cell extracts were immunoblotted to detect phosphorylated or total EGFR and GAPDH levels.

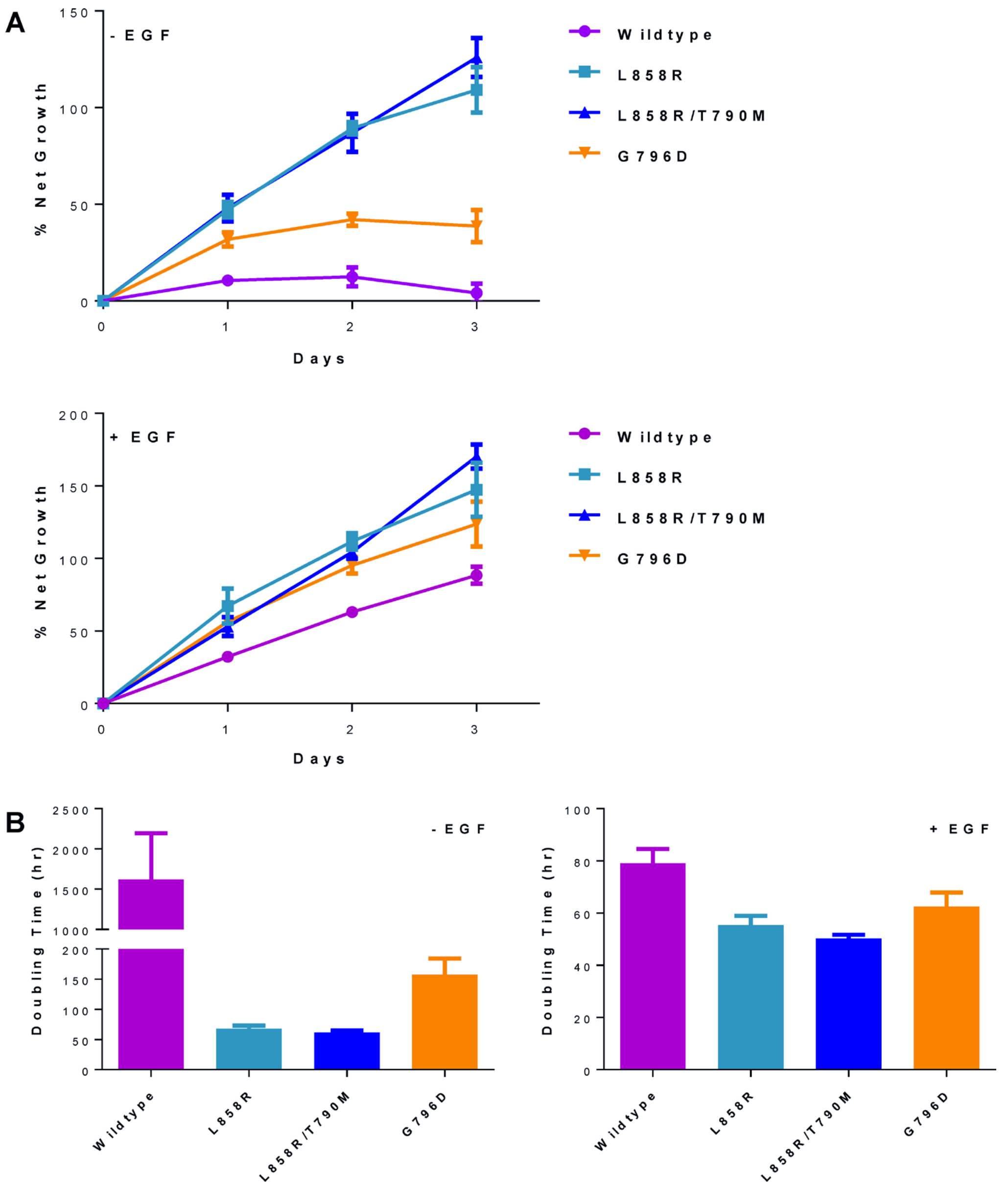

Supplementary Figure 3: EGFR G796D is a modest driver mutation. (**A**) Ba/F3 cells harboring various EGFR mutants were cultured in RPMI1640 + 10% FBS without IL3 nor EGF (upper) or with 10 ng/ml EGF supplemented (lower). Viable cells were measured every day and cell proliferation was calculated as % Net Growth = $(G_{day} - G_0)/G_{\mathit{0}}$ x 100%. Experiments were performed in 6 replicated wells, with mean ± S.D. plotted. (**B**) Doubling time was calculated as Doubling Time = [Duration × log(2)]/[log(Final Concentration) - log(Initial Concentration)]. Left: without EGF; Right: with 10 ng/ml EGF supplemented.

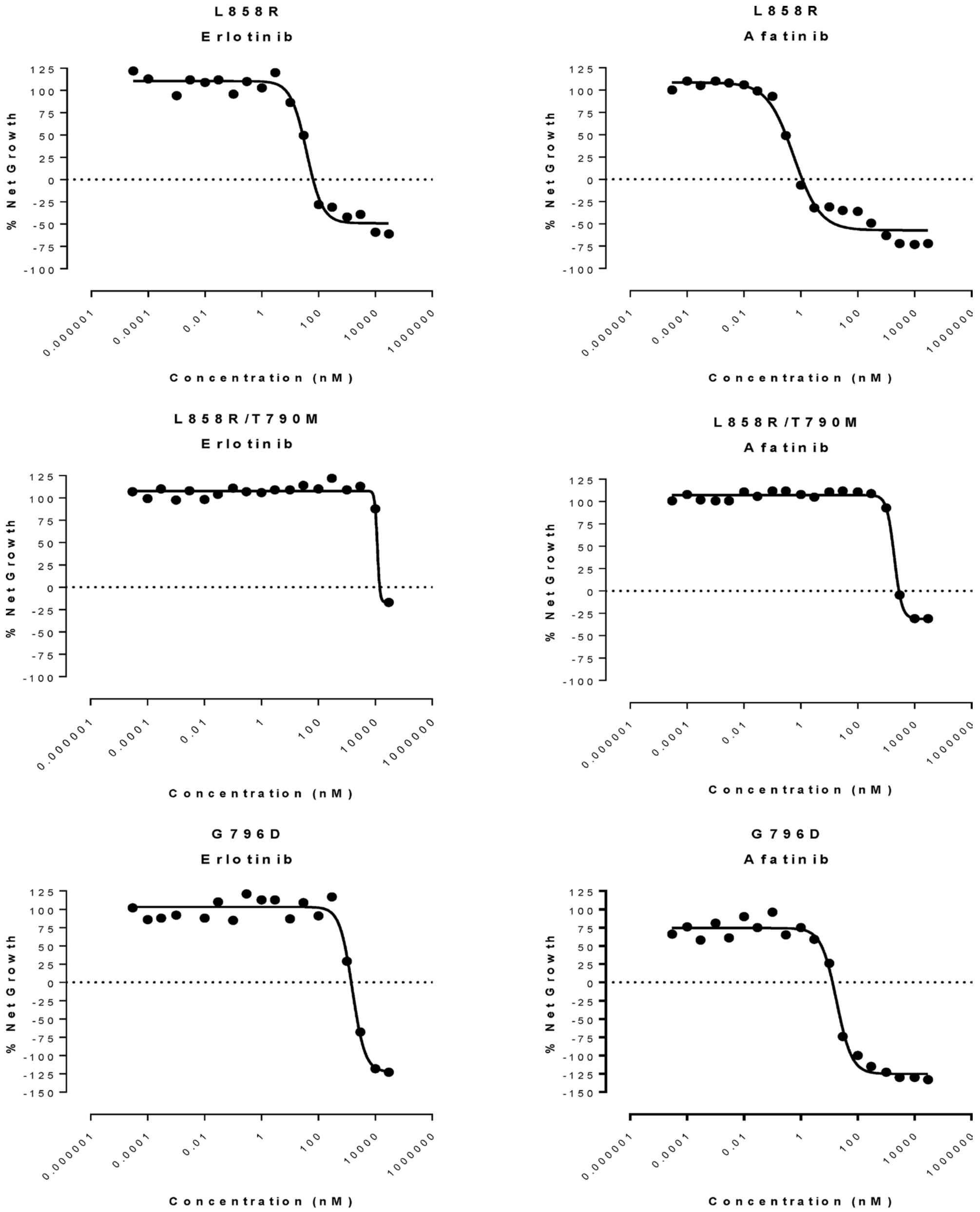

GI$_{50}$ (μM)	Erlotinib	Afatinib
**L858R**	0.03	0.0008
**L858R/T790M**	>10	2.71
**G796D**	0.71	0.01

Supplementary Figure 4: EGFR G796D is resistant to erlotinib but shows sensitivity to afatinib. Ba/F3 cells harboring L858R, L858R/T790M or G796D were treated with erlotinib or afatinib at indicated concentrations. Viable cells were measured after 72 h of treatment and cell proliferation was calculated as % Net Growth = $(G_{day3,\ inh} - G_0)/(G_{day3,\ ctl} - G_0) \times 100\%$. The curves were fitted using a nonlinear regression model with a sigmoidal dose response.

Supplementary Table 1: Genes included in the next-generation panel sequencing. See Supplementary_ Table_1.
