# Supplementary material for: EGFR G796D mutation mediates resistance to osimertinib
Source: Oncotarget. 2017 May 16;8(30):49671–9. doi: 10.18632/oncotarget.17913 (PMC5564797; doi:10.18632/oncotarget.17913)
Supplement: Supplementary file 2 [file oncotarget-08-49671-s002.doc]

**Supplementary Table 1: Genes included in the next-generation panel sequencing**

| **394 genes (whole exons)** | | | | | |
| --- | --- | --- | --- | --- | --- |
| ABCA6 | CDKN1A | FGF9 | LRRK2 | PAMR1 | RNF43 |
| ABCB1 | CDKN1B | FGFR1 | LYRM5 | PARP1 | RPA1 |
| ABL1 | CDKN2A | FGFR3 | MALAT1 | PAX6 | RPA2 |
| ACVR1B | CDKN2B | FGFR4 | MAP2K1 | PBRM1 | RPA3 |
| ACVR2A | CDKN2C | FHOD3 | MAP2K2 | PCBP1-AS1 | RPL22 |
| ACVRL1 | CEBPA | FIP1L1 | MAP2K4 | PDPK1 | RPL5 |
| ADAM28 | CHD7 | FKBP1A | MAP3K1 | PGM5 | RPL6 |
| AGTR2 | CHEK1 | FLT3 | MAP3K13 | PHF6 | RPSAP58 |
| AKT1 | CHEK2 | FOXA1 | MAP3K8 | PHLPP1 | RUNX1 |
| AKT1S1 | CLVS1 | FOXA2 | MAPK1 | PHLPP2 | SBNO1 |
| AKT2 | CNTLN | FOXC1 | MAPK3 | PIK3C2B | SDK1 |
| AKT3 | COL6A3 | FOXL2 | MAPK8IP1 | PIK3C2G | SETBP1 |
| AMER1 | CR2 | FRMD4A | MAPKAP1 | PIK3C3 | SETD2 |
| AOC3 | CRIPAK | FRS2 | MBD6 | PIK3CA | SF3B1 |
| APC | CRKL | GAS6 | MCL1 | PIK3CB | SH3KBP1 |
| AR | CRYGD | GATA3 | MDC1 | PIK3CD | SIN3A |
| ARAF | CSNK2A1 | GATA3-AS1 | MDM2 | PIK3CG | SLC27A3 |
| ARHGAP35 | CTCF | GLI1 | MDM4 | PIK3R1 | SMAD2 |
| ARID1A | CTNNB1 | GNA11 | MECOM | PIK3R2 | SMAD4 |
| ARID1B | CYP2D6 | GNA13 | MED12 | PIM1 | SMC1A |
| ARID2 | DCUN1D1 | GNAQ | MEDAG | PIM2 | SMC3 |
| ARID5B | DDC | GNAS | MFRP | PIM3 | SMIM4 |
| ASXL1 | DDR1 | GNG12 | MGA | PLEKHA6 | SOX17 |
| ATM | DDR2 | GSE1 | MITF | PMS1 | SOX9-AS1 |
| ATP2A1 | DEPTOR | GSTP1 | MLH1 | PMS2 | SPOP |
| ATP6V1B1 | DIS3 | H2AFX | MLH3 | POLD1 | STAG2 |
| ATR | DNMT3A | H3F3C | MLST8 | POLE | STK11 |
| ATRX | DOCK2 | HGF | MPL | POLM | SVIL |
| AXIN2 | DSG3 | HIST1H1C | MRAS | POLQ | TAF1 |
| B2M | DYRK4 | HIST1H2BD | MRE11A | PPOX | TBL1XR1 |
| ADGRB3 | EDNRB | HNF1A | MSH3 | PPP2R1A | TBX3 |
| BAP1 | EGR3 | HRAS | MSH6 | PPP2R2A | TBX4 |
| BARD1 | EIF4A2 | IDH1 | MTHFR | PRKAA1 | TDP1 |
| BCHE | ELF3 | IDH2 | MTOR | PRKAA2 | TET2 |
| BCL2L1 | EP300 | IGF1R | MUC16 | PRKAB1 | TEX26-AS1 |
| BCL2L11 | EPCAM | IL32 | MUTYH | PRKDC | TFDP1 |
| BCL9 | EPHA3 | IL6 | MVK | PRPF40B | TGFBR2 |
| BCORL1 | EPHB6 | INF2 | MYC | PRX | TLR4 |
| BMPR1A | EPPK1 | INPP4A | MYCL | PTCH1 | TMEM132D |
| BRAF | ERAS | INPP4B | MYCN | PTEN | TP53 |
| BRD2 | ERBB2 | IRAK4 | MYH9 | PTENP1 | TP53BP1 |
| BRD3 | ERBB3 | IRS4 | MYOF | PTPN11 | TPTE |
| BRD4 | ERBB4 | JAK1 | NAV3 | PTPN6 | TRAM1L1 |
| BRD9 | ERCC1 | JAK2 | NBN | RABGAP1 | TSHZ2 |
| BRIP1 | ERCC2 | JAK3 | NCOR1 | RAC1 | TSHZ3 |
| BUB1B | ERRFI1 | JARID2 | NCOR2 | RAD21 | TTC19 |
| BZRAP1 | ESR1 | KDM5C | NDUFB11 | RAD50 | U2AF1 |
| BZRAP1-AS1 | EZH2 | KDM6A | NDUFB2 | RAD51 | USP9X |
| C6orf89 | FAM175A | KDR | NF1 | RAD51AP2 | UTP23 |
| C7orf50 | FANCI | KEAP1 | NF2 | RAD51B | VCAN |
| C9orf131 | FANCL | KIF7 | NFE2L2 | RAD51C | VEZF1 |
| CBFB | FAT4 | KIT | NFE2L3 | RAD51D | VHL |
| CBL | FBXW7 | KLC3 | NKX2-1 | RAD51L3-RFFL | WBP1 |
| CCND1 | FGF1 | KLF3 | NKX2-8 | RAD52 | WNT16 |
| CCND2 | FGF10 | KLLN | NLK | RAD54L | WT1 |
| CCND3 | FGF12 | KMT2B | NOTCH1 | RAF1 | XPC |
| CCNE1 | FGF14 | KMT2C | NPM1 | RALGAPB | XRCC1 |
| CD4 | FGF19 | KMT2D | NRAS | RASA1 | XRCC2 |
| CDC14A | FGF2 | KRAS | NRG1 | RB1 | XRCC4 |
| CDH1 | FGF23 | KRTAP5-5 | NSD1 | RBM10 | XRCC5 |
| CDH10 | FGF3 | LAMP1 | NTRK3 | RELN | XRCC6 |
| CDK12 | FGF4 | LARP4B | OR5M3 | RHEB | XYLT2 |
| CDK4 | FGF5 | LIFR | OTX2 | RHOA | ZBTB20 |
| CDK6 | FGF6 | LIG1 | PAFAH1B1 | RICTOR | ZNF43 |
| CDK8 | FGF7 | LIG4 | PAK1 | RIT1 |  |
| CDK9 | FGF8 | LMAN1 | PALB2 | RNF2 |  |
| **9 genes (whole exons and introns)** | | | | | |
| ALK | BRCA2 | FGFR2 | MSH2 | RET | ROS1 |
| BRCA1 | EGFR | MET |  |  |  |
